# Effects of dietary stinging nettle (*Urtica dioica*) on hormone stress and selected serum biochemical parameters of broilers subjected to chronic heat stress

**DOI:** 10.1002/vms3.721

**Published:** 2022-01-12

**Authors:** Mehrad Mirsaiidi Farahani, Seyedeh Alemeh Hosseinian

**Affiliations:** ^1^ Department of Clinical Science School of Veterinary Medicine Shiraz University Shiraz Iran

**Keywords:** broiler, cortisol, heat stress, parameters, serum biochemical, stinging nettle

## Abstract

**Background:**

Heat stress is one of the most critical problems confronting the poultry industry. Stinging nettle (SN) is a medicinal plant with potent antioxidant properties.

**Objective:**

The goal of this study was to evaluate the effects of dietary SN at two different levels (2 and 4%) on the serum levels of cortisol and some selected parameters of broilers exposed to chronic heat stress.

**Methods:**

A total of 240 broiler chickens were randomly assigned to six dietary groups as follows: (1) control: fed the basal diet; (2) HS: heat‐stressed broiler fed the basal diet; (3) HS‐SN2: heat‐stressed broiler fed 2% dietary SN; (4) HS‐SN4: heat‐stressed broilers fed 4% SN; (5) SN2: no heat‐stressed broilers fed the basal diet supplemented with 2% SN; (6) SN4: no heat‐stressed broilers fed the basal diet supplemented with 4% SN. Diet supplementation with SN was performed from days 14 to 35 and chronic heat stress was induced from days 22 to 29. The serum parameters were evaluated on days 14, 21, 29 and 35.

**Results:**

HS had higher serum levels of cortisol, total cholesterol (TC), aspartate aminotransferase, alanine aminotransferase and creatine kinase (CK) compared to the other treatments. HS‐SN4 had significantly lower cortisol, TC, alanine aminotransferase and CK compared to HS and HS‐SN2.

**Conclusions:**

The inclusion of 4% SN powder in the broilers’ diet alleviated the negative effects of heat stress by decreasing cortisol, TC and tissue damage indices. It seems that dietary SN could be used as a feed additive in the poultry diet for improving the health status and defence mechanisms of the birds under stressful conditions.

## Graphical abstract

The present study investigated antioxidant activity of stinging nettle on chronic heat stress in broilers. Based on obtained results, it may be indicated that the addition of 4% stinging nettle powder to the broilers’ diet improved the oxidative status in heat‐stressed birds. Furthermore, this herb could be utilized as a feed additive in poultry diet to improve bird's health and defence mechanisms under stressful conditions.

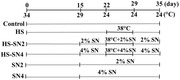

## 1 INTRODUCTION

Heat stress is one of the most prevalent environmental stressors affecting growth and impeding efficient poultry production (Souza‐Junior et al., 2019). It is an imbalance between the amount of heat production and heat loss in the bird's body (Zhang et al., 2012; Saracila et al., 2021). Broiler chickens are more sensitive to high ambient temperatures due to the rapid body metabolic rate and absence of sweat glands (Lara & Rostagno, 2013). Heat stress causes impairment in the welfare and health status of broilers. Furthermore, it leads to increased mortality and reduced body weight, feed intake and feed efficiency in the broilers (Olfati et al., 2018). Exposure to high environmental temperature leads to an increase in the respiratory rate and alters the homeostasis and acid‐base balance of the blood, leading to increases in the blood pH and incidence of respiratory alkalosis in broilers (Beckford et al., 2020). Exposure to chronic heat stress activates the hypothalamic—pituitary–adrenal axis, leading to an increased production of cortisol in broilers chickens (Quinteiro‐Filho et al., 2017). Therefore, blood biochemical assessment is a valuable tool to monitor the health situation and determine the severity of the heat stress in heat‐stressed (HS) broilers (Dapeng et al., 2020).

Blood biochemical parameters are reliable indicators of the well‐being of birds and reflect the nutritional, physiological and pathological status of birds’ body (Kraus et al., 2021). Thus, some serum indices provide clinical information for diagnosis and characterization of avian diseases (Hosseinian and Hasanzade, 2021; Li et al., 2017). As mentioned below, high ambient temperature causes several changes in the physiological status and blood biochemical profile in broilers (Wasti et al., 2020). Several researchers stated that some serum parameters are employed to determine oxidative stress and metabolic status of the broilers exposed to heat stress (Hosseini‐Vashan & Raei‐Moghadam, 2019). It has been confirmed that the serum activity of alanine aminotransferase (ALT) and aspartate aminotransferase (AST) enzymes were higher in broilers exposed to heat stress than the birds at optimum ambient temperature (Chand et al., 2018). Heat stress causes marked changes in serum levels of cholesterol, total protein (TP) and uric acid (UA) (Chand et al., 2018). Assessing the serum level of cortisol is another valuable indicator in various stressful conditions including heat stress in birds (Dapeng et al., 2020). Attenuating the adverse effects of heat stress is a valuable objective of the poultry industry. Therefore, various studies have applied different dietary antioxidants to ameliorate the harmful effects of heat stress in broilers (Khan et al., 2012). Various antioxidant compounds and antioxidant‐rich plants were added as natural feed additives to the diet to improve health status, antioxidant property and productive performance of birds under exposure to stressful conditions (Hosseinian & Ansari, 2021; Luo et al., 2018). Stinging nettle (SN) (*Urtica dioica*) is a medicinal plant that is employed as a phytogenic feed additive in the poultry diet (De Vico et al., 2018).

*Urtica dioica L*. (Urticaceae), commonly known as nettle or SN, is a perennial plant utilized as a traditional medicinal plant in numerous countries in the world (Mueen & Parasuraman, 2016). SN contains a high amount of terpenoids, phenylpropanoids, flavonol glycosides, carotenoids, tocopherol and ascorbic acid, which contribute to the anti‐inflammatory, antioxidant, anti‐viral and analgesic activities of nettle's leaves (Kregiel et al., 2018). Fresh leaves of SN or their extracts were added to broilers’ diet in some parts of Europe as a feed additive (Loetscher et al., 2013). Kregiel et al. (2018) reported that SN had positive effects on improving well‐being and health status in broilers. It has been demonstrated that supplementary SN at levels 2–4% improved the growth performance and body's antioxidant capacity in broilers (Sharma et al., 2018). Ahmadipour and Khajali (2019) applied the dietary supplementation of SN powder to attenuate the adverse effects induced by pulmonary hypertension syndrome.

There is no information about the effects of SN on serum metabolic characteristics in HS broilers. Therefore, the present study was designed to evaluate the effects of dietary SN at two different levels (2 and 4%) on the serum levels of metabolic indices and cortisol in chronic HS broilers. The results of the current study may aid in the better dietary management of broilers under exposure to heat stress.

## 2 MATERIALS AND METHODS

### 2.1 Birds, diets and experimental groups

On day 1, 240 1‐day‐old male *Arbor Acres* broiler chicks were randomly placed in stainless‐steel cages (200 cm length, 100 cm width, 80 cm height) with 10 birds/m^2^ density, along with one feed trough (185 cm long, 8.5 cm wide and 5.3 cm deep) and six nipple drinkers in thermostatically controlled rooms. All the nutritional and environmental conditions were programmed based on *A. Acres* Broiler Management Guide (Aviagen, 2018).

During the experiment, all the broilers had unrestricted access to a commercial starter (1–10 days old), grower (11–25 days old) and finisher (26–35 days old) diets. All the diets were formulated based on corn and soybean meal to meet the nutrient recommendations of the broilers, according to National Research Council (NRC, 1994) as described in Table 1. All the birds were reared in similar states for adaptation and fed by a similar basal diet until the 14 days of age.

**TABLE 1:** Ingredients and chemical composition of basal diets (as fed basis)

Items	Diets		
	Starter	Grower	Finisher
Ingredients (gram/100 gram, as fed)			
Yellow corn	53.4	58.0	61.3
Soybean meal (44%)	39.0	35.0	31.0
Di‐calcium phosphate	1.7	1.5	1.3
CaCO_3_ (38%)	1.7	1.4	1.3
Sunflower oil	3.0	3.0	4.0
Sodium chloride	0.3	0.3	0.3
Methionine	0.2	0.15	0.15
Lysine	0.2	0.15	0.15
Premix*	0.5	0.5	0.5
Chemical composition			
ME (kcal/kg)	2900	3000	3100
Crude protein (%)	22	20.5	19
Calcium (%)	1.05	0.9	0.8
Available Phosphorus (%)	0.5	0.45	0.4
Sodium (%)	0.19	0.19	0.19
Lysine (%)	1.31	1.25	1.14
Methionine (%)	0.54	0.48	0.44
Threonine (%)	0.85	0.81	0.75
Tryptophan (%)	0.25	0.22	0.2

* Vitamin and mineral content per kilogram of premix: vitamin A: 3,600,000 IU; vitamin D3: 800,000 IU; vitamin E: 7200 IU; vitamin K3: 0.8 g; vitamin B1: 0.71 g; vitamin B2: 2.64 g; vitamin B3: 3.92 g, vitamin B5: 11.88 g; vitamin B6: 1.176 g; vitamin B12: 6 mg; folic acid: 0.4 g; biotin: 40 mg; choline chloride: 100 g; selenium: 80 mg; cobalt: 100 mg; iodine: 396 mg; copper:4 g; zinc: 33.88 g; iron: 20 g; manganese: 39.68 g.

On day 14, the birds were randomly allotted into six equal groups of 40 chicks each and four replicates of 10 birds. The treatment groups are as follow: the first group (control): fed by basal diet and reared under normal ambient temperature (24 ± 1°C), the second group (HS): fed by basal diet and reared under cyclic heat stress (38 ± 1°C), the third and fourth groups (HS‐SN2 and HS‐SN4): birds reared under heat stress and fed by basal diet supplemented with 2 and 4% SN powder, respectively, and the fifth and sixth groups (SN2 and SN4): birds reared under normal ambient temperature and fed by basal diet supplemented with 2 and 4% SN powder, respectively.

All the birds were fed the basal diet from days 1 to 14. Afterwards, from days 14 to 35, SN powder was added to the basal diet in the treatment groups (HS‐SN2, HS‐SN4, SN2 and SN4). In this study, dried stems and leaves of SN were purchased from a local medicinal herb shop, identified by a botanist and were used. The qualitative and quantitative analysis of several active compounds in SN powder was performed by gas chromatography–mass spectrometry (GC‐MS) using Agilent 7890B GC system with Agilent 5977 Mass Selective Detector) (Table 2).

**TABLE 2:** Analysis of components of used stinging nettle (%)

Components	Content
Carvacrol	37.89
Naphthalene	8.9
Carvone	8.39
Butyldiene phthalide	6.1
Anethol	3.8
Phytol	3.31
Hexahydrofarnesyl acetone	3.13
Linalool	2.51
(E)‐Geranyl acetone	2.49
Furanone	2.45
Caryophyllene oxide	2.19
Cumin aldehyde	2.23
β‐caryophyllene	1.87
Bisabolene	1.87
Pentyl benzene	1.65
Cadinene	1.64
Limonene	1.50
Methyl chavicol	1.42
Kessane	1.15
Eugenol	0.98
Nonanal	0.86
Calamenene	0.81
β‐selinene	0.76
α‐terpineol	0.56
Cadina	0.48
Linalyl acetate	0.46
2‐pentyl furan	0.33
Copaene	0.27

### 2.2 Heat stress challenge

**FIGURE 1:**
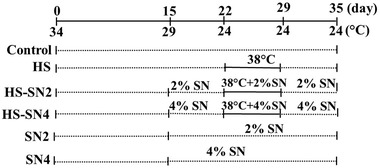
Experimental schedule of chronic heat‐exposure method. HS: heat‐stressed broiler; HS‐SN2: heat‐stressed broiler fed by 2% dietary stinging nettle; HS‐SN4: heat‐stressed broilers fed by 4% stinging nettle; SN2: No exposure to heat stress and fed by 2% dietary stinging nettle; SN4: No exposure to heat stress and fed by 4% dietary stinging nettle; SN: stinging nettle

On day 1, the ambient temperature was set as 34 ± 1°C and continued for the first 3 days. Then, room temperature gradually decreased 1°C every 2 days until reaching to 24 ± 1°C and maintained at this temperature by controlling heat and ventilation until day 22. On day 22, the ambient temperature suddenly increased from 24 ± 1 to 38 ± 1°C in the HS groups (HS, HS‐SN2, and HS‐SN4). The cyclic heat stress (38 ± 1°C) was induced with a heater from days 22 to 29 and the relative humidity was kept at 50 ± 5% during this period (Altan et al., 2003). The HS groups were reared in the controlled room at 38 ± 1°C for 8 h/D (between 09:00 AM and 5:00 PM), followed by 24°C for the remaining 16 h/D. The control, SN2 and SN4 groups were kept in separated rooms under normal temperature (24 ± 1°C) for 24 h/D. During the heat stress period, the fans were kept active to maintain the homogeneity of the environmental temperature in room space. Afterwards, on day 29, the room temperature suddenly decreased from 38 ± 1 to 24 ± 1°C in the HS groups, all the treatment groups were kept under ambient temperature (24 ± 1°C) until 35 days of age (Figure 1).

### 2.3 Blood biochemical assay

Blood sample (4 mL) was taken from the brachial vein from eight birds in each group on days 14, 21, 29 and 35. Following the blood coagulation at room temperature, the blood samples were centrifuged at 3000 × *g* for 10 min to obtain serum. Subsequently, sera were carefully collected and stored at −20°C until biochemical analysis. The levels of serum TP, total cholesterol (TC) and UA were measured using an automated analyser with commercial clinical investigation kits (Biorexfars kit, Fars, Iran). The serum concentration of cortisol was evaluated by the ELISA technique using the commercial kits (DIMETRA, Italy) according to the instructions of the manufacturer of the kit. The activities of AST, ALT and creatine kinase (CK) were measured with commercial kits (Biorexfars kit) based on the manufacturer's instructions.

### 2.4 Statistical analyses

Statistical analyses were carried out using SPSS (SPSS for Windows, Version 22, SPSS Inc., Chicago, Illinois). All the data obtained were subjected to one‐way ANOVA followed by the post‐hoc Tukey HSD test. The results were expressed as mean ± standard error (mean ± SE). Repeated measures ANOVA was employed for determining the effects of sampling time, differences between groups and interactions between time × treatments followed by Tukey's post‐hoc comparison test. Values of *p *< 0.05 were considered statistically significant.

## 3 RESULTS

Table 3 presents the effects of dietary SN on the serum levels of cortisol, TP, cholesterol and UA in the different groups of broilers subjected to heat stress. The concentrations of cortisol, TP, TC and UA on days 14 and 21 had no statistically significant differences among the studied groups (Table 3; *p *> 0.05). Heat stress increased the circulating levels of cortisol, TC and UA in the HS group compared to the control on days 29 and 35 (Table 3; *p *< 0.05). On days 29 and 35, SN supplementation at both levels of 2 and 4% reduced significantly cortisol, TC and UA levels in the HS broilers in the HS‐SN2 and HS‐SN4 groups compared to HS and these effects were stranger in HS‐SN4 group (Table 3; *p *< 0.05). There were no statistically significant differences in UA between the control group with HS‐SN2, HS‐SN4, SN2and SN4 groups on day 35 (Table 3; *p *> 0.05). The serum level of TP had no significant differences between the various treatments on days 29 and 35 (Table 3; *p *> 0.05).

**TABLE 3:** Effects of dietary stinging nettle on the serum levels of cortisol, total protein, cholesterol and uric acid (mean ± SE) in various groups of broilers subjected to chronic heat stress

		Age (days)				*p*‐value		
Indices	Groups	14	21	29	35	Group	Days	Group × days
Cortisol (ng/ml)	Control	1.36 ± 0.27^a^	1.44 ± 0.22^a^	1.44 ± 0.25^a^	1.49 ± 0.24^a^	<0.001	<0.001	<0.001
	HS	1.41 h ± 0.24^a^	1.75 ± 0.08^a^	3.59 ± 0.25^b^	2.76 ± 0.21^b^			
	HS‐SN2	1.47 ± 0.20^a^	1.30 ± 0.26^a^	2.97 ± 0.29^c^	2.22 ± 0.24^b^			
	HS‐SN4	1.44 ± 0.19^a^	1.36 ± 0.25^a^	2.18 ± 0.14^c^	1.61 ± 0.25^a^			
	SN2	1.43 ± 0.24^a^	1.22 ± 0.19^a^	1.63 ± 0.25^a^	1.50 ± 0.23^a^			
	SN4	1.41 ± 0.18^a^	1.43 ± 0.19^a^	1.48 ± 0.14^a^	1.46 ± 0.24^a^			
Total protein (g/dl)	Control	3.09 ± 0.22^a^	2.94 ± 0.22^a^	2.83 ± 0.12^a^	2.84 ± 0.31^a,b^	<0.001	<0.001	<0.001
	HS	2.99 ± 0.21^a^	3.42 ± 0.21^a^	2.47 ± 0.21^a^	2.84 ± 0.18^a,b^			
	HS‐SN2	2.55 ± 0.21^a^	3.23 ± 0.39^a^	2.88 ± 0.17^a,b^	3.28 ± 0.31^a,b^			
	HS‐SN4	2.99 ± 0.22^a^	3.27 ± 0.22^a^	3.33 ± 0.26^b^	3.58 ± 0.26^a^			
	SN2	2.69 ± 0.18^a^	3.20 ± 0.23^a^	3.49 ± 0.23^b^	2.91 ± 0.18^a,b^			
	SN4	3.17 ± 0.25^a^	3.07 ± 0.26^a^	3.29 ± 0.27^b^	2.69 ± 0.31^b^			
Cholesterol (mg/dl)	Control	130.30 ± 3.63^a^	137.05 ± 10.01^a^	127.02 ± 8.52^a^	135.11 ± 6.85^a^	<0.001	<0.001	<0.001
	HS	129.40 ± 3.46^a^	149.32 ± 6.15^a^	149.51 ± 6.65^b^	134.19 ± 7.26^a^			
	HS‐SN2	133.10 ± 2.84^a^	133.11 ± 10.80^a^	109.21 ± 10.21^a^	103.75 ± 7.02^b^			
	HS‐SN4	129.90 ± 3.50^a^	143.23 ± 11.26^a^	125.20 ± 7.75^a^	125.21 ± 7.57^a,b^			
	SN2	128.30 ± 4.87^a^	132.47 ± 9.72^a^	128.78 ± 5.22^a^	135.81 ± 6.03^a^			
	SN4	130.60 ± 2.02^a^	132.11 ± 8.83^a^	113.21 ± 10.85^a^	115.55 ± 10.42^b^			
Uric acid (mg/dl)	Control	3.65 ± 0.22^a^	3.22 ± 0.29^a^	2.80 ± 0.15^a^	3.04 ± 0.16^a^	<0.001	<0.001	<0.001
	HS	3.17 ± 0.23^a^	2.97 ± 0.15^a^	6.80 ± 0.24^b^	3.62 ± 0.20^b^			
	HS‐SN2	2.58 ± 0.18^b^	2.87 ± 0.06^a^	4.45 ± 0.24^c^	3.23 ± 0.18^a,b^			
	HS‐SN4	3.25 ± 0.28^a^	2.71 ± 0.12^a^	3.98 ± 0.22^c^	3.39 ± 0.28^a,b^			
	SN2	3.04 ± 0.25^a^	2.96 ± 0.07^a^	2.69 ± 0.19^a^	2.87 ± 0.14^a^			
	SN4	3.32 ± 0.30^a^	3.01 ± 0.13^a^	3.05 ± 0.19^a^	3.35 ± 0.17^a,b^			

Control: basal diet at normal temperature; HS: basal diet at cyclic heat stress; HS‐SN2: basal diet supplemented by 2% stinging nettle at cyclic heat stress; HS‐SN4: basal diet supplemented by 4% stinging nettle at cyclic heat stress; SN2; basal diet supplemented by 2% stinging nettle at normal temperature; SN4: basal diet supplemented by 4% stinging nettle at normal temperature.

Different letters in the superscripts of the same columns indicate significant differences (*p *< 0.05).

As shown in Table 4, the serum activities of AST and CK had no significant differences among the various studied groups on days 14 and 21 (Table 4; *p *> 0.05). On days 29 and 35, AST, ALT and CK were significantly higher in HS than the other experiment treatments (Table 4; *p *< 0.05). HS‐SN2 and HS‐SN4 had significantly lower serum activities of ALT and CK compared with HS on days 29 and 35. At the different days of study, the least serum levels of AST, ALT and CK were seen in control, SN2 and SN4 groups (Table 4; *p *< 0.05).

**TABLE 4:** The effects of stinging nettle on the serum activities levels of enzymes related to tissue damage in broilers exposed to chronic heat stress

		Age (days)				*p*‐value		
Indices	Groups	14	21	29	35	Group	Days	Group × days
ALT (U/L)	Control	78.25 ± 3.41^a,b^	82.17 ± 3.64^a^	75.99 ± 4.78^a^	78.60 ± 4.40^a^	<0.001	<0.001	<0.001
	HS	72.60 ± 3.50^a^	72.21 ± 5.08^a^	137.21 ± 3.62^b^	98.58 ± 3.46^b^			
	HS‐SN2	82.54 ± 3.69^a,b^	81.51 ± 3.61^a^	106.09 ± 4.60^c^	69.92 ± 4.10^a^			
	HS‐SN4	76.63 ± 9.04^a,b^	74.41 ± 4.36^a^	92.25 ± 2.48^d^	73.29 ± 4.61^a^			
	SN2	89.12 ± 3.03^b^	75.62 ± 4.38^a^	69.42 ± 4.35^a^	77.50 ± 3.74^a^			
	SN4	78.23 ± 4.52^a,b^	81.41 ± 2.41^a^	74.59 ± 4.65^a^	69.29 ± 2.22^a^			
AST (U/L)	Control	206.10 ± 17.86^a^	203.97 ± 19.92^a^	204.42 ± 9.04^a^	209.50 ± 18.25^a^	<0.001	<0.001	<0.001
	HS	241.09 ± 28.52^a^	195.74 ± 18.20^a^	359.57 ± 25.74^b^	276.84 ± 21.89^b^			
	HS‐SN2	207.51 ± 18.67^a^	230.33 ± 23.38^a^	297.61 ± 29.38^b^	252.61 ± 31.17^a,b^			
	HS‐SN4	214.96 ± 22.80^a^	225.91 ± 30.60^a^	298.83 ± 31.60^b^	249.32 ± 23.47^a,b^			
	SN2	203.54 ± 34.78^a^	242.25 ± 28.83^a^	203.35 ± 18.44^a^	200.91 ± 17.38^a^			
	SN4	191.22 ± 26.45^a^	203.97 ± 19.89^a^	208.69 ± 23.81^a^	196.43 ± 24.21^a^			
CK (U/L)	Control	1350.33 ± 143.84^a^	1361.52 ± 115.09^a^	1367.67 ± 172.33^a^	1285.09 ± 148.36^a^	<0.001	<0.001	<0.001
	HS	1235.71 ± 130.59^a^	1277.44 ± 135.67^a^	2160.14 ± 203.75^b^	1485.78 ± 129.22^a^			
	HS‐SN2	1307.43 ± 141.85^a^	1345.67 ± 138.72^a^	1489.63 ± 208.66^a^	1328.62 ± 181.71^a^			
	HS‐SN4	1354.59 ± 136.26^a^	1444.19 ± 140.85^a^	1398.11 ± 164.77^a^	1322.88 ± 125.24^a^			
	SN2	1323.28 ± 152.67^a^	1309.72 ± 122.44^a^	1313.18 ± 133.77^a^	1531.73 ± 140.76^a^			
	SN4	1394.31 ± 129.18^a^	1232.34 ± 123.61^a^	1278.64 ± 125.82^a^	1323.58 ± 140.96^a^			

AST, aspartate aminotransferase; ALT, alanine aminotransferase; CK, creatine kinase; Control, basal diet at normal temperature; HS, basal diet at cyclic heat stress; HS‐SN2, basal diet supplemented by 2% stinging nettle at cyclic heat stress; HS‐SN4, basal diet supplemented by 4% stinging nettle at cyclic heat stress; SN2; basal diet supplemented by 2% stinging nettle at normal temperature; SN4: basal diet supplemented by 4% stinging nettle at normal temperature.

Different letters in the superscripts of the same columns indicate significant differences (*p *< 0.05).

## 4 DISCUSSION

Heat stress is a major environmental stressor with a negative impact on poultry well‐being and health status. Various studies have stated that heat stress causes oxidative stress, resulting in a change in the blood biochemical stress parameters (Habashy et al., 2017). Several nutritional methods have been assessed to attenuate the adverse effects of heat stress (Attia et al., 2017). Diet supplementation with medicinal plants is one of the most common solutions to alleviate heat stress in broilers (Chauhan et al., 2020; Sărăcilă et al., 2020). Accordingly, the current research assessed the effects of dietary SN on some blood biochemical metabolites in broilers exposed to heat stress. This study revealed that dietary SN powder at a high dose (4%) minimizes the chronic heat stress in broilers by normalizing the serum values of cortisol, TC and UA (Table 3). Furthermore, dietary SN at 4% level decreased the serum activities of ALT, AST and CK enzymes as tissue damage indices in the birds exposed to chronic heat stress (Table 4). Hence, SN could be added to the broilers’ diet as a feed additive to combat the negative effects of stressful conditions.

Blood biochemical assessment is a valuable tool to monitor the bird's health situation and is the basis of disease diagnosis (Dolka et al., 2014). Alteration in serum parameters indicates a change in metabolism and biochemical processes of the bird, resulting from the effects of various infectious and non‐infectious conditions (Vinkler et al., 2010). Antioxidant compounds can alter blood biochemical parameters in broilers under stressors due to free‐radical neutralizing, inhibiting lipid peroxidation and activating antioxidant enzymes (Horváth & Babinszky, 2018). SN has significant antioxidant effects due to its phenolic and terpenoid compounds (Kregiel et al., 2018). Carvacrol and carvone are the main terpenoids in this herb with antioxidant and growth‐promoting properties (Upton, 2013). In the present experiment, the content of carvacrol and carvone in the SN powder was 46.28% of the oil (Table 2). In agreement with these findings, Ahmadipour and Khajali (2019) demonstrated that SN powder with potent antioxidant capacity contained 44.8% carvacrol and carvone. In this regard, the effects of dietary SN on the serum levels of cortisol, TP, TC, UA, ALT, AST and CK‐MB were evaluated in the broilers subjected to chronic heat stress (Tables 3 and 4).

In the current study, the concentrations of cortisol, TP, cholesterol, UA, ALT, AST and CK on days 14 and 21 had no statistically significant difference among the examined groups, implying no stress on the birds before exposure to heat stress (Table 3; *p *> 0.05). Following chronic heat stress, the serum levels of cortisol significantly increased in HS compared to the control reared under normal environmental temperature on days 20 and 35. These findings are in agreement with previous studies stating that chronic heat stress increases the serum level of cortisol in broilers (Xu et al., 2018). In the current experiment, both dietary levels of SN in the HS‐SN2 and HS‐SN4 groups reduced the cortisol compared with the HS group on the basal diet on days 29 and 35; however, this effect was stranger at high dose (4%) of SN supplementation (Table 3; *p *< 0.05). Based on the current investigation, 4% dietary SN led to a significant decrease in the serum level of cortisol, indicating that this herb could attenuate the stress response induced by heat exposure in broilers.

Herein, the serum levels of TP had no significant differences among the various groups (Table 3; *p *> 0.05). Several studies have reported that the TP level increased in birds following inflammation or stressors (Kim et al., 2015; Shen et al., 2010). Furthermore, Sugiharto et al. (2017) stated that heat stress resulted in an increase in the TP in broilers. Interestingly, no significant differences were was observed in the TP concentration between the different studied treatments (Table 3). Similarly, some other researchers have found no significant alterations in the TP in birds exposed to heat stress (Kataria et al., 2008; Vosmerova et al., 2010).

In the studied birds, heat stress led to an increase in TC concentration in HS compared with the control group. These findings are in line with the results by Luo et al. (2018). They confirmed that heat stress decreased the activity of lipolytic enzymes and the rate of lipolysis in the body, which led to an increase in serum levels of triglyceride and TC. In the present experiment, the serum level of cholesterol decreased in the HS‐SN2, HS‐SN4 and SN‐4 groups fed by supplemented diet with SN. The cholesterol reduction activity of SN could depend on its sterol and phenolic content (Kregiel et al., 2018). Moreover, SN reduces lipogenesis and lipid metabolism, resulting in a reduction in the serum content of TC (Avci et al., 2006). In agreement with the obtained findings from this study, Zhang et al. (2020) demonstrated that the inclusion of SN in the diet of the laying hen was associated with the decreased TC and triglycerides. Based on the results presented in Table 3, dietary supplementation with SN at level 4% had positive and significant effects on decreasing the circulating cholesterol level and improving the physiological response of the broilers during heat exposure and 7 days following the exposure to heat stress. It could be concluded that supplementing the basal diet with 4% SN can protect birds against adverse effects of heat stress.

In the present study, the serum level of UA was significantly higher in the HS group compared to the control group (Table 3; *p *< 0.05). UA is the end‐metabolic product of protein metabolism in avian species and has been suggested as a potent scavenger of free radicals in broiler chickens (Azzam et al., 2019). Xie et al. (2015) reported that the circulating level of UA increased in HS broilers and it could be utilized as a detectable indicator of oxidative stress. Furthermore, Qaid and Al‐Garadi (2021) stated that the serum concentration of UA increased in broilers under exposure to heat stress to neutralize free radicals. In accordance with these results, the UA content of serum increased in the HS group compared to the birds at normal ambient temperature (control) (Table 3). Lobo et al. (2010) stated that uric acid had antioxidant activity and a powerful effect on reducing the free radicals in the body. Due to the reduction in cortisol, cholesterol and UA in the studied broilers, it could be concluded that supplementing the diet with 4% SN improved the physiological state biochemical parameters in the broilers under chronic HS condition.

Based on presented results in Table 4, heat stress increased (*p *< 0.05) the serum activities of ALT, AST and CK in the HS group compared with the control group, on days 29 and 35. Chronic thermal exposure leads to the over‐production of free radicals and subsequent oxidative damage in the various body tissues (Akbarian et al., 2016). The liver negatively affected by chronic heat stress in birds (Miao et al., 2020). The serum levels of ALT and AST enzymes are employed as reliable indices of liver damage and can reflect the degree of oxidative damage in the hepatic cells during exposure to chronic heat stress (Miao et al., 2020; Xie et al., 2015). Xie et al. (2015) reported that chronic heat stress increased the serum activities of AST, and CK enzymes, which is in agreement with the obtained findings of this study. Furthermore, heat stress significantly increased the serum activity of CK, as a valuable indicator for muscular damage induced by chronic heat stress (Xie et al., 2015), which is consistent with the results in Table 4. The serum activities of ALT and CK decreased in the HS broilers fed by 2 and 4% dietary SN (HS‐SN2 and HS‐SN4, respectively) compared with HS (Table 4, *p *< 0.05). These results are in line with the previous studies stating that SN had hepato‐protective property (Uyar et al., 2016). SN inhibits liver damage with its potent antioxidant activity (Yildizhan et al., 2020). In the current experiment, the effects of SN on alleviating the hepatic damage were revealed with the decreases in the serum level of ALT and CK in the broilers. The obtained results from the current study indicated that SN regulates the oxidative stress induced by heat stress in broilers and protects the hepatic cells from damage in the birds exposed to heat stress.

## 5 CONCLUSIONS

Heat stress exposure caused an elevation in circulating cortisol as a main stress indicator. Furthermore, the serum levels of TC, UA and activities of ALT, AST and CK increased in HS broilers. The supplemented diet with SN at a level of 4% decreased the serum levels of cortisol, cholesterol, AST, ALT and CK in the broilers exposed to chronic heat stress. Accordingly, it seems that dietary SN attenuates the negative effects of HS in broilers. In addition, it could be suggested that SN be applied as a feed additive for improving the health status of broilers under stressful conditions.

## AUTHORS’ CONTRIBUTION

Mehrad Misaedi: Conceptualization, funding acquisition, methodology, visualization, writing—original draft.

Seyyedeh Alemeh Hosseinian: Conceptualization, data curation, formal analysis, funding acquisition, investigation, methodology, project administration, resources, software, supervision, validation, visualization, writing—review & editing

## AUTHOR DECLARATION

All of the authors listed on the manuscript are employed by a government agency (Shiraz University) that has not a primary function other than research and/or education.

## ANIMAL ETHICAL STATEMENT

All protocols used in the study were approved by the Iranian Animal Ethics Standards under the supervision of the Iranian Society for the prevention of cruelty to animals and Shiraz University Research Council (IACUC no: 4687/63).

## CONFLICT OF INTEREST

The authors declare no conflict of interest.

## Data Availability

Research data are not shared.
